# Nasopharyngeal SARS-CoV-2 Load at Hospital Admission as a Predictor of Mortality

**DOI:** 10.1093/cid/ciaa956

**Published:** 2020-07-16

**Authors:** Claudia Alteri, Valeria Cento, Marta Vecchi, Luna Colagrossi, Diana Fanti, Chiara Vismara, Massimo Puoti, Carlo Federico Perno, SCoVA Study Group, Claudia Alteri, Maria Antonello, Chiara Baiguera, Alessandra Bielli, Maurizio Bottiroli, Paolo Brioschi, Daniela Campisi, Stefania Carta, Giorgia Casalicchio, Valeria Cento, Arturo Chieregato, Luna Colagrossi, Valentino Costabile, Jacopo Colombo, Federica Di Ruscio, Oscar Massimiliano Epis, Diana Fanti, Roberto Fumagalli, Thomas Langer, Elisa Matarazzo, Marco Merli, Alice Nava, Silvia Nerini Molteni, Carlo Federico Perno, Massimo Puoti, Silvia Renica, Livia Tartaglione, Nicola Ughi, Chiara Vismara

**Affiliations:** 1 Department of Oncology and Hemato-oncology, University of Milan, Milan, Italy; 2 Residency in Microbiology and Virology, University of Milan, Milan, Italy; 3 Residency in Infectious Diseases, University of Milan, Milan, Italy; 4 Department of Laboratories, Bambino Gesù Children’s Hospital, Rome, Italy; 5 Chemical-Clinical and Microbiological Analyses, ASST Grande Ospedale Metropolitano Niguarda, Milan, Italy; 6 Infectious Diseases, ASST Grande Ospedale Metropolitano Niguarda, Milan, Italy

to the editor—We read with interest the article by Bhargava et al [1] that was recently published in the Journal, which significantly contributes to the definition of clinical risk factors for severe coronavirus disease 2019 (COVID-19) manifestations. However, despite the comprehensive analysis of multiple clinical and laboratory parameters, the virus is still a poorly represented piece of this puzzle. During the severe acute respiratory syndrome coronavirus (SARS-CoV-1) epidemic, higher viral loads were strongly correlated with disease severity and death [2]. Similarly, the newly emerged severe acute respiratory syndrome coronavirus 2 (SARS-CoV-2) demonstrated a strikingly fast, and intense, replication kinetics, whose contribution to the clinical evolution of COVID-19 is starting to be investigated [3–6]. The nasopharyngeal SARS-CoV-2 load expressed by cycle thresholds (Cts) of real-time polymerase chain reaction (RT-PCR), the standard-of-care for COVID-19 molecular diagnosis [7, 8], is a widely available parameter to be correlated with the severity of COVID-19. To prove this hypothesis, we investigated the correlation between the initial nasopharyngeal SARS-CoV-2 loads and 30-day in-hospital mortality in 206 consecutive adult patients with a laboratory-confirmed SARS-CoV-2 infection, admitted to Niguarda Hospital (Milan, Italy) since 5 March, and who have either died or been discharged by 23 April 2020 (study protocol: 92-15032020). Dynamic ranges of categorized Ct values of viral RdRp, E, and N genes were assessed by quantitative droplet-digital PCR.

The median (interquartile range) time from symptom onset to hospital admission was 6 (4–9) days. By then, 188 of 206 (91.3%) patients presented an interstitial pneumonia with ground-glass opacities. Survivors (n = 153) and nonsurvivors (n = 53) significantly differed in several anamnestic, clinical, virological, and laboratory characteristics (Supplementary Appendix). These included both the mean and the absolute Ct values of RdRp, N, and E genes that were significantly lower in nonsurvivors compared with survivors (*P* = .001), reflecting higher viral loads in the nasal/throat compartment of the former patients. Of note, 20.7% of nonsurvivors had initial Cts less than 20 (viral loads ≥10^7^ copies/mL) versus only 5.3% of survivors (*P* < .001).

Kaplan-Meier curves showed a progressive increase in 30-day mortality, by increase in nasopharyngeal SARS-CoV-2 load (Figure 1). Thirty days after disease onset, the survival rate dropped to 35.3% in patients with Cts less than 20 (viral loads ≥10^7^ copies/mL) versus 81.0% in patients with Cts greater than 35 (viral loads <10^3^ copies/mL) (*P* = .001). Notably, the 36.4% of patients with initial Cts of less than 20 died within 7 days versus 14.3% and 0.0% of patients with initial Cts of 20–24.9 and higher (Cts >25) (*P* = .006).

**Figure 1.**
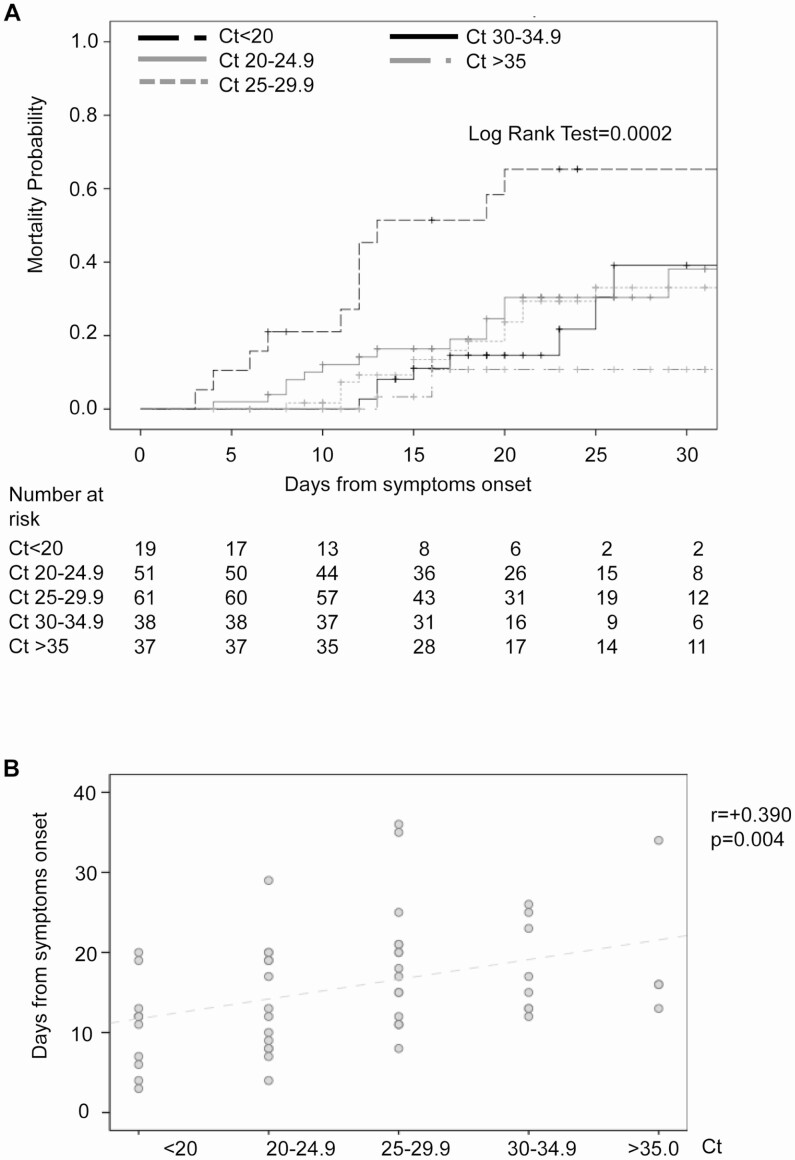
Contribution of nasopharyngeal SARS-CoV-2 shedding to in-hospital mortality. *A*, Kaplan-Meier estimates of mortality probability within the first 30 days from disease onset, according to nasopharyngeal Ct values at admission. *B*, Nasopharyngeal Ct values at admission against time of mortality from disease onset (days) in nonsurvivors. The Pearson correlation coefficient between Ct values and time of mortality is displayed in the top-right corner of the panel (*r* = +0.390). Abbreviations: Ct, cycle threshold; SARS-CoV-2, severe acute respiratory syndrome coronavirus 2.

In Cox proportional hazards models (Supplementary Appendix), out of 19 variables analyzed, Cts less than 20 remained one of the strongest predictors of in-hospital death, both in univariate (hazard ratio [HR], 8.38; 95% confidence interval [CI]: 2.66–26.37; *P* = .00015) and in multivariate analysis (HR, 3.94; 95% CI: 1.75–8.87; *P* = .001) (along with presence of comorbidities, creatinine, D-dimers, and C-reactive protein).

Overall, we identified the high initial nasopharyngeal viral load as an independent risk factor for in-hospital mortality, and for a significantly faster worsening of clinical conditions towards death. These results strengthen the recently reported correlation between viral load and severe disease [5, 6] and provide initial evidence of a role for viral load in influencing the definitive outcome. As RT-PCR on nasopharyngeal swabs is used worldwide, clinically validated Ct cutoffs (ie, <20) represent a ready-to-use prognostic marker to help stratify patients for risk of in-hospital death, and to consequently implement appropriate measures to contain fatalities.

## Supplementary Data

Supplementary materials are available at *Clinical Infectious Diseases* online. Consisting of data provided by the authors to benefit the reader, the posted materials are not copyedited and are the sole responsibility of the authors, so questions or comments should be addressed to the corresponding author.

ciaa956_suppl_Supplementary_Appendix

## Notes

***The SCoVA*** (***S**equenicing **CO**ronavirus and **V**ariability **A**nalysis*) ***Study Group:*** Claudia Alteri, Maria Antonello, Chiara Baiguera, Alessandra Bielli, Maurizio Bottiroli, Paolo Brioschi, Daniela Campisi, Stefania Carta, Giorgia Casalicchio, Valeria Cento, Arturo Chieregato, Luna Colagrossi, Valentino Costabile, Jacopo Colombo, Federica Di Ruscio, Oscar Massimiliano Epis, Diana Fanti, Roberto Fumagalli, Thomas Langer, Elisa Matarazzo, Marco Merli, Alice Nava, Silvia Nerini Molteni, Carlo Federico Perno, Massimo Puoti, Silvia Renica, Livia Tartaglione, Nicola Ughi, Chiara Vismara.

***Acknowledgments.*** The authors thank the Collaborator Members of the **S**equenicing **CO**ronavirus and **V**ariability **A**nalysis (SCoVA) Study Group. The authors also thank all of the staff of the Microbiology and Virology Laboratory of ASST Grande Ospedale Metropolitano Niguarda for their outstanding technical support in processing swab samples and performing laboratory analyses and data management.

***Disclaimer.*** The funding source had no role in the study design; in the collection, analysis, and interpretation of data; in the writing of the report; or in the decision to submit the paper for publication.

***Financial support.*** This work was supported by the Italian Ministero dell’Istruzione · Ministero dell’Università e della Ricerca (MIUR), Ministry of Education, University and Research (PRIN grant number 20179JKAMZ).

***Potential conflicts of interest.*** The authors: No reported conflicts of interest. All authors have submitted the ICMJE Form for Disclosure of Potential Conflicts of Interest.
