# Supplementary material for: Nasopharyngeal SARS-CoV-2 Load at Hospital Admission as a Predictor of Mortality
Source: Clin Infect Dis. 2020 Jul 16;72(10):1868–9. doi: 10.1093/cid/ciaa956 (PMC7454479; doi:10.1093/cid/ciaa956)
Supplement: ciaa956_suppl_Supplementary_Appendix [file ciaa956_suppl_supplementary_appendix.docx]

**Appendix Table 1. Demographic, clinical, viral, laboratory, and imaging findings at hospital admission, of 206 patients with COVID-19**

|  | **Overall, N=206** | **Non-survivors, N=53** | **Survivors, N=153** | **P-value** |
| --- | --- | --- | --- | --- |
| **Demographics and clinical characteristics** |  |  |  |  |
| Age, years | 64 (51-74) | 73 (68-79) | 59 (49-70) | <0.001 |
| Sex |  |  |  |  |
| *Male* | 139 (67.5) | 45 (84.9) | 94 (61.4) | 0.002 |
| Systolic blood pressure, mmHg | 130 (120-145) | 135 (120-150) | 130 (120-145) | 0.146 |
| *>140 mmHg* | 57 (27.7) | 18 (34.0) | 39 (25.5) | 0.15 |
| Chronic comorbidities | 128 (62.1) | 44 (83.0) | 84 (54.9) | <0.001 |
| *Hypertension* | 72 (35.0) | 30 (56.6) | 42 (27.5) | <0.001 |
| *Obesity* | 23 (11.3) | 7 (13.2) | 16 (10.6) | 0.385 |
| *Diabetes* | 32 (15.5) | 17 (32.1) | 15 (9.8) | <0.001 |
| *Cardiovascular disease* | 25 (12.1) | 13 (24.5) | 12 (7.8) | 0.002 |
| *Chronic obstructive lung disease* | 22 (10.7) | 11 (20.8) | 11 (7.2) | 0.009 |
| *Malignancies* | 30 (14.6) | 11 (20.8) | 19 (12.4) | 0.106 |
| *Chronic kidney disease* | 14 (6.8) | 5 (9.4) | 9 (5.9) | 0.275 |
| *Chronic liver disease* | 8 (3.9) | 0 (0.0) | 8 (5.2) | 0.088 |
| *Othe*r^*^ | 6 (2.9) | 1 (1.9) | 5 (3.3) | 0.515 |
| Symptoms |  |  |  |  |
| *Fever* | 186 (90.3) | 48 (90.6) | 138 (90.2) | 0.767 |
| *Temperature at presentation,°C* | 37.5 (36.9-38.1) | 37.7 (37-38.3) | 37.5 (36.8-38) | 0.516 |
| *Cough* | 134 (65.0) | 29 (54.7) | 105 (68.6) | 0.128 |
| *Dyspnea* | 97 (47.1) | 32 (60.4) | 65 (42.5) | 0.027 |
| *Time since dyspnea onset, days* | 2 (1-4) | 2 (1-5) | 2 (1-3) | 0.992 |
| *Sputum* | 5 (2.4) | 2 (3.8) | 3 (2) | 0.592 |
| *Myalgia* | 35 (17.0) | 5 (9.4) | 30 (19.6) | 0.134 |
| *Rhinorrhea* | 2 (1.0) | 0 (0.0) | 2 (1.3) | 1 |
| *Headache* | 7 (3.4) | 3 (5.7) | 4 (2.6) | 0.373 |
| *Sore throat* | 1 (0.5) | 0 (0.0) | 1 (0.7) | 1 |
| *Gastrointestinal symptoms^†^* | 24 (11.7) | 6 (11.3) | 18 (11.8) | 1 |
| *Neurological symptoms^‡^* | 6 (2.9) | 3 (5.7) | 3 (2.0) | 0.178 |
| Time from symptoms-onset to hospital admission, days | 6 (4-9) | 5 (3-6) | 6 (4-9) | 0.003 |
| Time from symptoms-onset to final outcome, weeks | 2.7 (1.9-3.7) | 2.1 (1.6-2.9) | 2.9 (1.1-4.1) | <0.001 |
| **SARS-CoV-2 RT-PCR** |  |  |  |  |
| Positive*^§^* | 177 (85.9) | 50 (94.3) | 127 (83.0) | 0.041^\|\|^ |
| Weakly positive^¶^ | 29 (14.1) | 3 (5.7) | 26 (17) |  |
| RdRp cycle thresholds | 28.7 (25.3-34.7) | 27.2 (23.6-30.5) | 29.9 (26-35.6) | 0.001 |
| E cycle thresholds | 26.4 (22.9-31) | 24.8 (20.3-28.2) | 27.5 (23.4-31.4) | 0.001 |
| N cycle thresholds | 26.1 (22.2-31.8) | 24.2 (19.7-28.7) | 27.3 (23.1-32.1) | 0.001 |
| Mean cycle thresholds^**^ | 27 (23.5-32.4) | 25.3 (21.4-28.9) | 28.3 (24.1-32.9) | 0.001 |
| *<20* | 19 (9.2) | 11 (20.7) | 8 (5.3) | 0.001^\|\|^ |
| *20-24.9* | 50 (24.3) | 13 (24.5) | 37 (24.2) |  |
| *25-29.9* | 62 (30.1) | 17 (32.1) | 45 (29.4) |  |
| *30-34.9* | 38 (18.4) | 8 (15.1) | 30 (19.6) |  |
| *>35* | 37 (22.1) | 4 (7.5) | 33 (21.6) |  |
| **Laboratory findings** |  |  |  |  |
| White blood cell count, × 10⁹ per L |  |  |  | 0.032^\|\|^ |
| *< 4* | 35 (17.0) | 5 (9.4) | 30 (19.6) |  |
| *4-10* | 151 (73.3) | 39 (73.6) | 112 (73.2) |  |
| *> 10* | 19 (9.2) | 9 (17.0) | 10 (6.5) |  |
| Haemoglobin, mg/dL | 13.3 (12.3-14.4) | 13.1 (12.3-14.6) | 13.4 (12.3-14.4) | 0.603 |
| Anemia | 5 (2.4) | 2 (3.8) | 3 (2.0) | 0.606 |
| Platelet count, × 10⁹ per L | 195 (159-257) | 183 (151-229) | 200 (162-268) | 0.045 |
| Albumin, g/L | 3.52 (3.18-3.88) | 3.29 (2.93-3.44) | 3.68 (3.4-4.01) | 0.002 |
| *< 100* | 9 (4.4) | 4 (7.5) | 5 (3.3) | 0.241 |
| ALT, U/L | 27 (18-42) | 25 (17-41) | 28 (18-42) | 0.559 |
| *> 40* | 57 (27.7) | 14 (26.4) | 43 (28.1) | 1 |
| AST, U/L | 33 (25-46) | 36 (28-59) | 31 (23-42) | 0.002 |
| *> 40* | 59 (28.6) | 22 (41.5) | 37 (24.2) | 0.03 |
| Total bilirubin, mg/mL | 0.6 (0.4-0.8) | 0.7 (0.4-1) | 0.5 (0.4-0.8) | 0.025 |
| *> 1* | 31 (15.0) | 14 (26.4) | 17 (11.1) | 0.013 |
| Estimated Glomerular Filtration Rate^††^, mL/min |  |  |  | 0.002^\|\|^ |
| *≥ 60* | 123 (82.6) | 19 (35.8) | 104 (88.1) |  |
| *30-59* | 18 (12.1) | 9 (29.0) | 9 (7.6) |  |
| *15-29* | 6 (4.0) | 3 (9.7) | 3 (2.5) |  |
| *< 15* | 2 (1.3) | 0 (0.0) | 2 (1.7) |  |
| Creatinine >133 μmol/L | 43 (20.9) | 23 (43.4) | 20 (13.1) | <0.001 |
| Prothrombin time, s | 13 (13-14) | 14 (13-15) | 13 (12.5-14) | <0.001 |
| *> 16* | 13 (6.3) | 10 (18.9) | 3 (2.0) | <0.001 |
| D-dimer*^‡‡^*, μg/mL | 0.97 (0.545-1.76) | 1.28 (0.83-2.14) | 0.89 (0.52-1.56) | 0.025 |
| *> 1* | 47 (55.3) | 17 (77.3) | 30 (47.6) | 0.024 |
| Fibrinogen, mg/dL | 550 (422.5-672) | 625 (413-708) | 540 (432-658) | 0.432 |
| Glycemia, mg/dL | 112 (99-135) | 120 (104-156) | 109 (99-124) | 0.009 |
| IL-6*^§§^*, pg/mL | 61 (28-137) | 153 (96-181) | 40.8 (24.3-101) | <0.001 |
| C-reactive protein, mg/L | 6 (2-12) | 11 (6-18) | 5 (2-10) | <0.001 |
| *> 8* | 86 (41.7) | 36 (67.9) | 50 (32.7) | <0.001 |
| Lactate dehydrogenase, U/L | 302 (237-389) | 402 (316-499) | 273 (224-325) | <0.001 |
| *> 245* | 117 (56.8) | 41 (77.4) | 76 (49.7) | <0.001 |
| **Imaging features**^\|\|\|\|^ |  |  |  |  |
| Ground-glass opacity | 188 (91.3) | 50 (94.3) | 138 (90.2) |  |
| Consolidation | 6 (2.9) | 0 (0.0) | 6 (3.9) |  |
| Lobar infiltration | 2 (1.0) | 1 (1.9) | 1 (0.7) |  |

Data are expressed as median (interquartile range), or N (%). P-values comparing non-survivors to survivors were calculated by Mann-Whitney U test, Chi^2^ test, or Fisher’s exact test, as appropriate. ALT=alanine aminotransferase. AST=aspartate transaminase. COVID-19, coronavirus disease 2019. E=envelope. IL-6=interleukin-6. N=nucleocapsid. RdRp=RNA-dependent RNA-polymerase. RT-PCR=real-time polymerase chain reaction.^*^Including: Crohn’s disease (n=1), Hashimoto’s thyroiditis (n=1), familial lipid disorders (n=2), rheumatoid arthritis (n=2). ^†^Including: diarrhea (N=6), nausea (N=1), abdominal pain (N=1). *^‡^*Including: ictus (N=1), syncope (N=2), epilepsy (N=1), hyposthenia (N=2). *^§^*Amplification successful for all 3 target genes (RNA-dependent RNA-polymerase, envelope and nucleocapsid), or for 2 out of 3 genes, but always with cycle thresholds<30. ^¶^Amplification successful for only 1 target gene, or for 2 out of 3, but with cycle thresholds ≥30. ^||^Chi^2^ test comparing all subcategories. ^**^Quantitative droplet-digital PCR on a subset of samples demonstrated that Ct <20, 20-24.9, 25-29.9, 30-34.9, >35 reflect a viral-load of ≥10^7^, <10^7^-≥10^6^, <10^6^-≥10^4^, <10^4^-≥10^3^, <10^3^ copies/mL, respectively. ^††^Information available for 149 individuals. *^‡‡^*Information available for 85 individuals. *^§§^*Information available for 77 individuals. ^||||^For the remaining 10 individuals (2 non survivors and 8 survivors) no lesions were detected.

**Appendix Table 2. Predictive value of cycle-threshold RT-PCR results for in-hospital death of SARS-CoV-2 infected patients**

|  | **Cox Univariate analysis** | | | **Cox Multivariate analysis** | | |
| --- | --- | --- | --- | --- | --- | --- |
|  | **HR** | **95%** | **P-value** | **HR** | **95%** | **P-value** |
| Age, years | 1.06 | 1.03-1.08 | 6.89E-06 | 1.02 | 0.99-1.05 | 0.212 |
| Sex |  |  |  |  |  |  |
| *Female* | 1 | Ref. | - | 1 | Ref. | - |
| *Male* | 2.63 | 1.24-5.59 | 0.012 | 0.53 | 0.25-1.14 | 0.11 |
| At least one comorbidity | 3.4 | 1.66-6.98 | 0.001 | 3.88 | 1.27-11.86 | 0.017 |
| Comorbidity (presence vs. absence) |  |  |  |  |  |  |
| *Hypertension* | 2.01 | 2.09-3.68 | 0.025 | 0.74 | 0.31-1.75 | 0.492 |
| *Diabetes* | 1.5 | 0.75-2.97 | 0.249 | 0.83 | 0.36-1.90 | 0.661 |
| *Cardiovascular disease* | 1.91 | 0.92-3.98 | 0.084 | 1.14 | 0.12-10.62 | 0.908 |
| *Chronic obstructive lung disease* | 2.43 | 1.12-4.90 | 0.024 | 0.7 | 0.05-9.12 | 0.783 |
| Presence of dyspnea | 1.46 | 0.84-2.55 | 0.182 | 1.97 | 0.97-4.02 | 0.061 |
| CT |  |  |  |  |  |  |
| >35 | 1 | Ref. | - | 1 | Ref. | - |
| 30-34.9 | 2.12 | 0.63-7.05 | 0.222 | 1.9 | 0.77-4.69 | 0.161 |
| 25-29.9 | 2.64 | 0.88-7.90 | 0.083 | 1.85 | 0.43-7.99 | 0.411 |
| 20-24.9 | 2.71 | 0.89-8.25 | 0.078 | 1.52 | 0.36-6.49 | 0.574 |
| <20 | 8.38 | 2.66-26.37 | 1.15E-04 | 3.94 | 1.75-8.87 | 0.001 |
| White blood cell count, × 10⁹ per L |  |  |  |  |  |  |
| >10 | 1 | Ref. | - | 1 | Ref. | - |
| 4-10 | 0.62 | 0.30-1.27 | 0.191 | 1.153 | 0.36-3.66 | 0.809 |
| < 4 | 0.49 | 0.16-1.47 | 0.204 | 0.389 | 0.08-1.93 | 0.249 |
| Platelet count, × 10⁹ per L | 0.99 | 0.98-0.99 | 0.006 | 0.99 | 0.98-1.00 | 0.19 |
| Total bilirubin, mg/mL | 1.45 | 1.06-1.97 | 0.018 | 1.31 | 0.80-2.16 | 0.28 |
| Creatinine, μmol/L | 4.11 | 2.37-7.12 | 4.33E-07 | 2.55 | 1.30-5.01 | 0.007 |
| *≤133* | 1 | Ref. | - | 1 | Ref. | - |
| *>133* | 4.11 | 2.37-7.12 | 4.33E-07 | 2.55 | 1.30-5.01 | 0.007 |
| Prothrombin time, s |  |  |  |  |  |  |
| *≤16* | 1 | Ref. | - | 1 | Ref. | - |
| *>16* | 4.37 | 2.17-8.79 | 3.56E-05 | 2.18 | 0.96-4.94 | 0.061 |
| D-dimer, μg/mL |  |  |  |  |  |  |
| *≤1* | 1 | Ref. | - | 1 | Ref. | - |
| *>1* | 2.75 | 1.01-7.46 | 0.047 | 1.14 | 1.06-1.24 | 0.001 |
| Glycemia, mg/dL | 1.53 | 0.61-3.87 | 0.38 | 1 | 0.99-1.00 | 0.451 |
| C-reactive protein, mg/L |  |  |  |  |  |  |
| *≤8* | 1 | Ref. | - | 1 | Ref. | - |
| *>8* | 2.45 | 1.37-4.38 | 0.002 | 1.11 | 1.04-1.17 | 0.001 |
| Lactate dehydrogenase, U/L |  |  |  |  |  |  |
| *≤245* | 1 | Ref. | - | 1 | Ref. | - |
| *>245* | 5.41 | 1.50-19.55 | 0.01 | 1.74 | 0.48-6.27 | 0.398 |

Relative hazard with 95% CI. Cox univariate and multivariate analyses are reported. Variables were excluded from univariate and multivariate analysis if available for less than 40% of patients (IL-6), if their between-group differences were not significant (Appedix Table 1), and if affected by collinearity (eGFR with Creatinine, AST with LDH). Ct=cycle threshold. HR=hazard ratio. RT-PCR=real-time polymerase chain reaction. ^*^RdRp Ct Univariate HR: 0.94 [CI:0.90-0.98], p=0.003 and Multivariate HR: 0.89 [CI:0.81-0.98], p=0.024; E Ct Univariate HR: 0.91 [CI:0.86-0.97], p=0.001 and Multivariate HR: 0.83 [CI:0.73-0.94], p=0.004; N Ct Univariate HR: 0.92 [CI:0.88-0.96], p=0.001 and Multivariate HR: 0.85 [CI:0.76-0.96], p=0.007.
